# Effect of inhaled anticholinergics on bronchial secretions: a systematic review

**DOI:** 10.3389/fphys.2026.1790151

**Published:** 2026-04-14

**Authors:** Leonardo Arzayus-Patiño, José Julián Bernal-Sanchez, Valeria Pérez-Hortua, Esther Cecilia Wilches-Luna, Vicente Benavides-Cordoba

**Affiliations:** 1 Facultad de Salud, Programa de Fisioterapia, Universidad Santiago de Cali, Cali, Colombia; 2 Grupo de Investigación Salud y Movimiento, Universidad Santiago de Cali, Cali, Colombia; 3 Facultad de Salud, Escuela de Rehabilitación Humana, Cali, Colombia; 4 Grupo de Investigación Ejercicio y Salud Cardiopulmonar (GIESC), Cali, Colombia; 5 Facultad de Salud, Departamento de Ciencias Fisiológicas, Universidad del Valle, Cali, Colombia

**Keywords:** bronchial secretions, cholinergic antagonists, inhaled anticholinergics, mucociliary clearance, sputum

## Abstract

**Background:**

Inhaled anticholinergics are commonly used in chronic respiratory diseases with the intention of reducing bronchial secretions in addition to their bronchodilatory effects. However, evidence regarding their direct influence on mucus production and clearance remains limited and fragmented. This study aimed to identify, evaluate, and synthesize the available evidence on the effects of inhaled and nebulized anticholinergics on mucus volume, rheological properties, mucociliary clearance, cough, and pulmonary function in adults.

**Methods:**

A systematic review was conducted following PRISMA 2020 guidelines and registered in PROSPERO (CRD42024575999). Comprehensive searches were performed in PubMed, OVID, Embase, SciELO, EBSCO, ScienceDirect, and Cochrane Library without date restrictions. Randomized and non-randomized studies assessing inhaled or nebulized anticholinergics were included. Risk of bias was evaluated using the RoB 2 and ROBINS-I tools.

**Results:**

Out of 2,449 records identified, eight studies comprising 403 participants met the inclusion criteria. Under stable clinical conditions, inhaled ipratropium and tiotropium showed no significant changes in sputum volume, viscosity, or mucociliary clearance compared with placebo. In acute or procedural settings, such as bronchoscopy, nebulized ipratropium was associated with a reduction in bronchial secretion grade and cough frequency. Tiotropium treatment demonstrated decreases in sputum solid fraction and mucin content (MUC5AC and MUC5B). None of the included studies reported impairment of mucociliary clearance or serious adverse events related to treatment.

**Conclusion:**

Current evidence does not demonstrate a consistent effect of inhaled or nebulized anticholinergics on sustained reduction of bronchial secretions. However, their use does not appear to cause adverse changes in mucus production or clearance and may induce transient effects during acute cholinergic stimulation. Overall, the findings support a favorable safety profile of these agents regarding airway secretion management.

**Systematic Review Registration:**

https://www.crd.york.ac.uk/PROSPERO/, identifier CRD42024575999.

## 1 Introduction

Mucus production in the central airways is under cholinergic control and plays a fundamental role in both physiological conditions and various chronic respiratory diseases (Thomas et al., 2010). Airway mucus acts as a protective barrier against inhaled noxious particles and contributes to the preservation of epithelial integrity; it is composed mainly of water, electrolytes, and mucins, glycoproteins responsible for its viscoelastic properties. Mucus secretion is carried out primarily by the submucosal glands and, to a lesser extent, by goblet cells of the respiratory epithelium, with submucosal glands being the main source of production regulated by the parasympathetic system. Acetylcholine, as the dominant neurotransmitter of the cholinergic system, acts on muscarinic receptors expressed in these structures, particularly M3 receptors, whose activation significantly increases mucus secretion in the bronchial airways. Although goblet cells may also respond to this stimulation, this generally occurs at higher concentrations of acetylcholine, which has led to the proposal that pharmacological blockade of muscarinic receptors could limit excessive secretion production in the context of exacerbations of chronic respiratory diseases (Rogers, 2007; Gosens and Gross, 2018; Balsamo et al., 2010).

Mucus hypersecretion in the airways refers to an abnormal increase in mucus production by the glands and secretory cells of the respiratory epithelium that exceeds the amount required for normal physiological function and may contribute to airway obstruction. In the respiratory context, this hypersecretion is mediated by chronic inflammation and by hyperplasia of goblet cells and submucosal glands, and is observed in diseases such as asthma, chronic obstructive pulmonary disease, and cystic fibrosis, where excessive mucus production not only obstructs the bronchial lumen but also promotes the development of mucus plugs and impairs secretion clearance (Fahy and Dickey, 2010).

Under physiological conditions, airway mucus exhibits viscoelastic properties that allow effective transport through mucociliary clearance and coughing. However, in settings of hypersecretion or chronic inflammation, these rheological properties, particularly viscosity and elasticity, may be significantly altered, increasing resistance to airflow and hindering the elimination of secretions. These alterations favor mucus retention, mucus plug formation, and mucociliary clearance dysfunction, directly contributing to airway obstruction and functional impairment observed in various chronic respiratory diseases (Rogers, 2004).

Drugs that regulate mucus can be classified as mucoactive agents, mucolytics, or mucoregulators. Among the latter are anticholinergics, which block parasympathetic nervous activity, thereby reducing mucus secretion. Anticholinergic medications may be administered intravenously or by inhalation. Among the best-known intravenous anticholinergics are atropine and scopolamine, whereas inhaled agents include ipratropium bromide, oxitropium, glycopyrrolate, and tiotropium. Intravenous atropine, often administered as atropine methonitrate, has been shown to block mucociliary clearance of the gel layer without altering the depth of the sol layer, although it is associated with well-known cardiac effects due to blockade of parasympathetic system function (Canning, 2006).

Inhaled anticholinergics are frequently used in patients with chronic respiratory diseases because of their bronchodilator effects. These medications block the action of the neurotransmitter acetylcholine at muscarinic receptors. Clinically used anticholinergics include natural, semisynthetic, and synthetic compounds that exhibit a wide range of actions on airway smooth muscle cells and the parasympathetic nervous system. Anticholinergics have antisecretory activity and reduce nasal and bronchial secretions as well as salivation, and are used to decrease secretions in allergic and inflammatory diseases (Barnes, 2000).

Ipratropium bromide, a short-acting synthetic derivative of atropine, is one of the most widely used anticholinergics. Both short-acting and long-acting anticholinergics, when administered by inhalation, produce bronchodilation and are widely approved for the treatment of chronic obstructive pulmonary disease and asthma. In addition, some animal studies have shown that these drugs reduce secretion production. Although they do not reduce normal secretion volume nor increase secretion viscosity, they do decrease hypersecretion triggered by inflammatory activation of muscarinic receptors (Barnes, 2001).

Despite the widespread use of inhaled anticholinergics in clinical practice (Papi et al., 2021), the specific effect of these drugs on bronchial secretions in humans has not been systematically synthesized. The available evidence is fragmented and derives from studies with heterogeneous designs, different methods for secretion assessment, and varied clinical contexts, which has contributed to the persistence of contradictory concepts regarding their true impact on mucus production, mucus characteristics, and airway clearance mechanisms.

Therefore, the objective of this systematic review was to identify, critically evaluate, and synthesize the available evidence on the effect of inhaled and nebulized anticholinergics on bronchial secretions in the adult population, including their impact on mucus volume and properties, mucociliary clearance, secretion-related cough, associated pulmonary function, and clinical safety.

## 2 Methods

### 2.1 Study design and registration

A systematic review of the literature was conducted following the recommendations of the PRISMA 2020 statement for systematic reviews. The review protocol was previously registered in the PROSPERO database (International Prospective Register of Systematic Reviews) under the registration number CRD42024575999 and was developed in accordance with the objectives and methods specified therein.

### 2.2 Research question

The research question guiding this review was: What is the effect of inhaled or nebulized anticholinergics on the volume, rheological properties, and clearance of bronchial secretions in humans?

The question was structured using the PICO framework as follows:

- P (Population): Adults (≥18 years) with respiratory diseases associated with alterations in bronchial secretions, such as chronic obstructive pulmonary disease, asthma, bronchiectasis, or other respiratory conditions.
- I (Intervention): Use of inhaled or nebulized anticholinergics, including short-acting and long-acting agents.
- C (Comparison): Placebo, no treatment, or other non-anticholinergic treatments (e.g., β_2_-agonists, corticosteroids, mucolytics).
- (Outcomes): Changes in bronchial secretions assessed through objective or functional measures, such as secretion volume, rheological properties of mucus (viscosity, elasticity), mucociliary clearance, cough efficacy associated with secretions, and related pulmonary function parameters. Safety outcomes were considered secondary endpoints.

### 2.3 Eligibility criteria

#### 2.3.1 Inclusion criteria

Studies meeting the following criteria were included:

- Randomized and non-randomized clinical trials, crossover studies, before–after studies, quasi-experimental studies, and observational studies.
- Studies conducted in adult humans.
- Studies evaluating anticholinergics administered via inhalation or nebulization.
- Studies that directly or indirectly measured outcomes related to bronchial secretions (volume, mucus characteristics, mucociliary clearance, secretion-related cough).
- Studies published in English, Spanish, or Portuguese.
- Studies published without date restrictions.

#### 2.3.2 Exclusion criteria

The following were excluded:

- Preclinical or animal studies.
- Studies evaluating anticholinergics administered exclusively via systemic routes.
- Studies combining inhaled anticholinergics with other drugs without the possibility of isolating their effect.
- Studies assessing only subjective expectoration outcomes without objective or functional measurements.
- Narrative reviews, editorials, letters to the editor, and case reports.

### 2.4 Information sources and search strategy

A systematic search was conducted in PubMed, OVID, Embase, SciELO, EBSCO, ScienceDirect, and the Cochrane Library. The literature search included studies published from the inception of each database until January 7, 2026, which corresponds to the date of the last update of the search strategy.

The search strategy combined controlled vocabulary (MeSH and DeCS) and free-text terms related to inhaled anticholinergics and bronchial secretions. No date filters were applied. Searches were updated prior to the final analysis to identify recently published studies.

The main terms used included combinations of controlled vocabulary (MeSH/DeCS) and free-text terms, organized into conceptual domains of population, intervention, comparison, and outcomes.

- Population: Humans, Adults, Adult, Middle Aged, Aged.
- Intervention (inhaled anticholinergics): Anticholinergic Agents, Muscarinic Antagonists, Ipratropium, Ipratropium Bromide, Tiotropium, Tiotropium Bromide, Glycopyrrolate, Aclidinium, Umeclidinium, Long-Acting Muscarinic Antagonists (LAMA), Short-Acting Muscarinic Antagonists (SAMA).
- Comparison: Placebo, Control Group, Usual Care, No Treatment, Standard Therapy, β2-Agonists, Bronchodilators, Corticosteroids, Mucolytics, Expectorants, Saline, Non-anticholinergic therapy.
- Outcomes (bronchial secretions and clearance mechanisms): Bronchial Secretions, Airway Mucus, Mucus Hypersecretion, Sputum, Sputum Volume, Mucus Viscosity, Mucus Elasticity, Mucins, MUC5AC, MUC5B, Mucociliary Clearance, Ciliary Function, Airway Clearance, Cough, Cough Frequency, Cough Clearance, Expectoration.

Terms were combined using Boolean operators (AND/OR) and adapted to the specific characteristics of each database. No publication date filters were applied.

Search equations were adapted for each database.

### 2.5 Study selection process

All records identified through the search were exported to Rayyan for management and duplicate removal. Two reviewers independently screened titles and abstracts according to the predefined eligibility criteria, and potentially relevant studies proceeded to full-text review. Disagreements at any stage of the selection process were resolved by consensus or, when necessary, with the involvement of a third reviewer. The study selection process was documented using a flow diagram in accordance with the PRISMA statement.

### 2.6 Data extraction

Data extraction was performed independently by two reviewers using a previously designed standardized form managed in Microsoft Excel. Data were extracted manually and cross-checked; discrepancies were resolved by consensus or, when necessary, with the involvement of a third reviewer. The following variables were extracted:

- Study identification (author, year, country).
- Study design and methodological characteristics.
- Sample size and population characteristics.
- Type of anticholinergic, dose, route of administration, and device.
- Comparator and co-interventions.
- Outcomes related to bronchial secretions, pulmonary function, cough, and safety.
- When information was not explicitly available, it was recorded as not reported.

### 2.7 Risk of bias assessment

The risk of bias of the included randomized clinical trials was assessed using the Cochrane Risk of Bias 2 (RoB 2) tool, which systematically evaluates domains related to the randomization process, deviations from intended interventions, incomplete outcome data, outcome measurement, and selection of the reported result (Sterne et al., 2019).

For non-randomized studies, the risk of bias was assessed using the Risk Of Bias In Non-randomized Studies of Interventions (ROBINS-I) tool, considering domains of bias due to confounding, selection of participants, classification of interventions, deviations from intended interventions, missing data, outcome measurement, and selection of the reported result (Sterne et al., 2016).

Risk of bias assessment was conducted independently by two reviewers, and any discrepancies were resolved by consensus or with the involvement of a third reviewer when necessary.

### 2.8 Synthesis of results

Due to the clinical and methodological heterogeneity of the included studies, results were synthesized primarily in a narrative manner. When possible, quantitative comparisons between groups or between pre- and post-intervention measurements were reported.

Studies were grouped according to the type of outcome evaluated: mucus volume and characteristics, mucociliary clearance, secretion-related cough, pulmonary function, and safety.

## 3 Result

### 3.1 Literature screening

**Figure 1:**
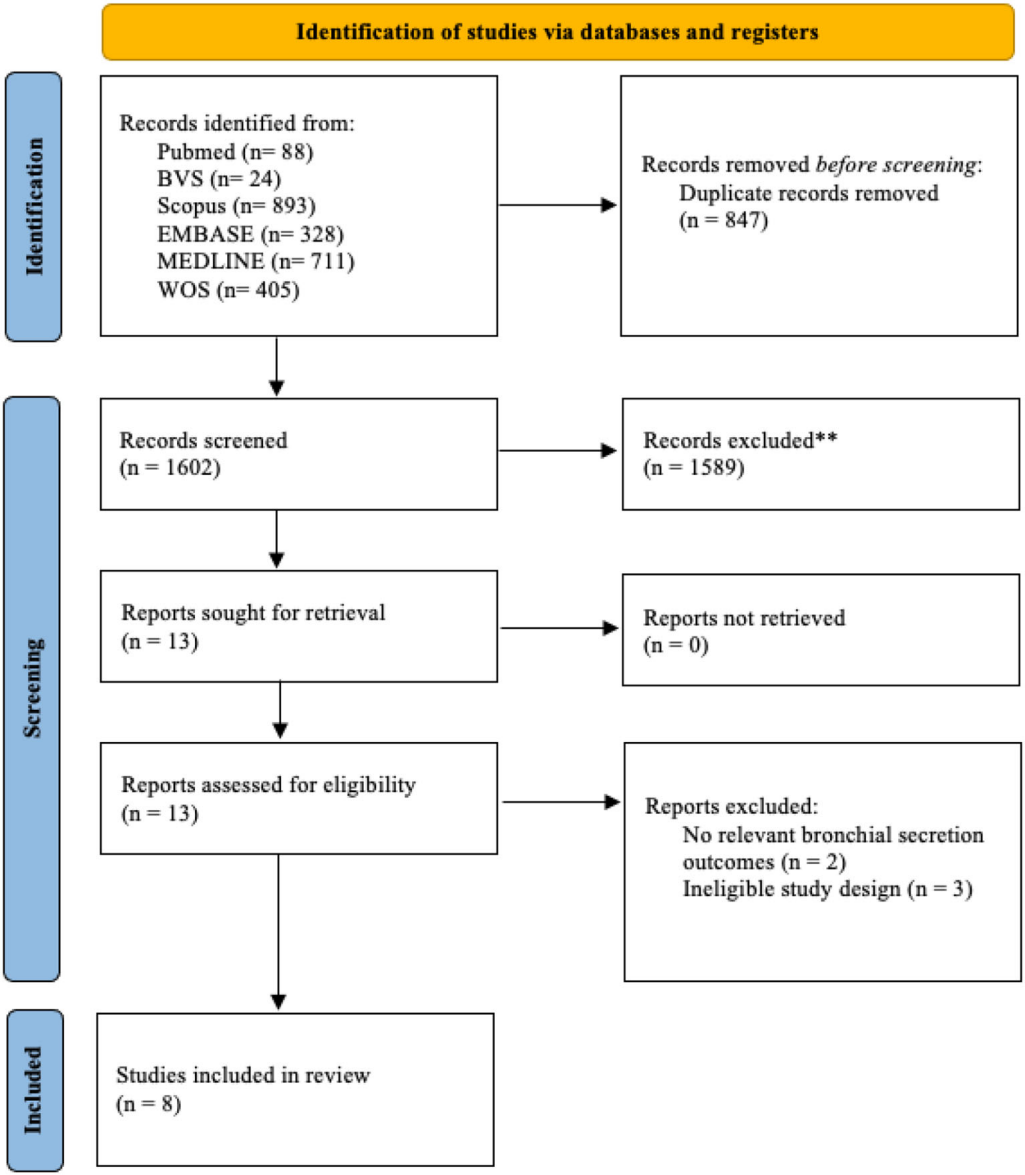
PRISMA flowchart of literature screening.

The systematic search identified 2,449 records across the consulted databases. After removal of 847 duplicates, 1,602 records were screened by title and abstract, of which 1,589 were excluded for not meeting the inclusion criteria. Subsequently, 13 studies were assessed in full text, and 5 were excluded for not reporting relevant outcomes related to bronchial secretions (n = 2) or for having a non-eligible methodological design (n = 3). Finally, 8 studies were included in the present systematic review (**Figure 1**).

### 3.2 General characteristics of the included studies

The eight studies included in this systematic review were published between 1979 and 2023 and were conducted in different geographical settings, including Europe (United Kingdom), Asia (Japan, China, and Indonesia), and the United States. The main characteristics of the studies, including country of origin, study design, study population, and inhaled anticholinergic interventions, are summarized in **Table 1**.

**Table 1:** Characteristics of the included studies.

Study ID	Country/location	Study design	Population	Sample size (n)	Condition/setting	Anticholinergic (dose, route)	Outcomes related to secretions
(Taylor et al., 1986)	United Kingdom	RCT, DB, CO	Adults with airway obstruction (asthma, COPD)	15	Stable obstructive airway disease	Ipratropium bromide, high dose, inhaled (MDI)	Mucociliary clearance; sputum volume; sputum viscosity; cough
(Pavia et al., 1979)	United Kingdom	RCT, DB, PC	Adults with chronic bronchitis and/or mild asthma	12	Reversible airway obstruction	Ipratropium bromide 40 µg, inhaled (MDI)	Mucociliary clearance; sputum weight; cough
(Bennett et al., 1993)	United States	RCT, DB, CO	Adults with moderate–severe COPD	15	Stable COPD	Ipratropium bromide, standard dose, inhaled (MDI)	Cough clearance; mucociliary clearance
(Hasani et al., 2004)	United Kingdom	RCT, DB, PC	Adults with moderate–severe COPD	34	Stable COPD	Tiotropium 18 µg/day, inhaled (DPI, HandiHaler^®^)	Mucociliary clearance; cough
(Gaffey et al., 1988)	United States	RCT, DB, PC	Healthy adult volunteers	69	Experimentally induced common cold	Ipratropium bromide 84 µg, intranasal spray	Nasal mucus production
(Tagaya et al., 2016)	Japan	Prospective BA	Adults with stable COPD	22	COPD with chronic cough and sputum	Tiotropium 18 µg/day, inhaled (DPI, HandiHaler^®^)	Sputum composition; mucociliary clearance; cough
(Wang et al., 2019)	China	RCT, DB, PC	Adults undergoing diagnostic bronchoscopy	250	Bronchoscopy under general anesthesia	Ipratropium bromide 1 mg, inhaled (nebulizer)	Bronchial secretions during procedure; cough
(Mutmainnah et al., 2023)	Indonesia	QE with control	Adults undergoing diagnostic bronchoscopy	36	Bronchoscopy	Ipratropium bromide 1 mg, inhaled (nebulizer)	Bronchial secretions; cough

RCT, randomized controlled trial; DB, double-blind; PC, placebo-controlled; CO, crossover; BA, before–after; QE, quasi-experimental.

Overall, the included studies evaluated approximately 403 participants, with sample sizes ranging from 12 to 250 subjects. The study populations included adults with chronic obstructive pulmonary disease, asthma, or reversible airway obstruction, as well as patients undergoing diagnostic procedures such as bronchoscopy and healthy volunteers in experimental settings. The characteristics of the populations evaluated are detailed in **Table 1**.

Regarding methodological design, randomized double-blind clinical trials were included (Pavia et al., 1979; Taylor et al., 1986; Bennett et al., 1993; Hasani et al., 2004; Wang et al., 2019), as well as crossover studies (Pavia et al., 1979; Taylor et al., 1986; Bennett et al., 1993) and quasi-experimental and prospective before–after studies (Tagaya et al., 2016; Mutmainnah et al., 2023). This methodological heterogeneity directly influenced the inability to perform a quantitative meta-analysis.

The most frequently evaluated anticholinergic was ipratropium bromide, used in six of the eight included studies (Pavia et al., 1979; Taylor et al., 1986; Gaffey et al., 1988; Bennett et al., 1993; Wang et al., 2019; Mutmainnah et al., 2023), whereas tiotropium was evaluated in two studies (Hasani et al., 2004; Tagaya et al., 2016). The predominant route of administration was inhalation, using pressurized metered-dose inhalers, dry powder inhalers, or nebulizers; however, one study evaluated intranasal ipratropium (Gaffey et al., 1988). The main characteristics of the interventions are summarized in **Table 2**.

**Table 2:** Secretion-related efficacy outcomes of included studies.

Author (year)	Anticholinergic (drug, dose, route)	Method for secretion assessment	Main secretion-related findings	Authors’ main conclusion
(Taylor et al., 1986)	Ipratropium bromide, high dose, inhaled	Radioaerosol technique (AUC 0–6 h); sputum weight and viscosity	No significant changes in mucociliary clearance, sputum volume, or sputum viscosity compared with placebo	High-dose inhaled ipratropium does not impair mucociliary clearance or alter sputum properties
(Pavia et al., 1979)	Ipratropium bromide 40 µg, inhaled	Radioaerosol mucociliary clearance; sputum weight and radioactivity	No change in global mucociliary clearance; reduced sputum weight	Ipratropium is an effective bronchodilator without adverse effects on mucociliary clearance
(Bennett et al., 1993)	Ipratropium bromide, inhaled, single dose	Radiolabeled particle clearance during cough	Reduced cough clearance efficiency with no change in basal mucociliary clearance	Ipratropium may reduce cough efficiency as a secretion clearance mechanism in COPD
(Hasani et al., 2004)	Tiotropium 18 µg/day, inhaled	Radioaerosol technique (AUC 0–6 h)	No delay in mucociliary clearance; increased peripheral aerosol penetration	Tiotropium does not adversely affect mucociliary clearance in COPD
(Gaffey et al., 1988)	Ipratropium bromide 84 µg, intranasal	Nasal mucus weight measurement	Reduced total nasal mucus production compared with placebo	Intranasal ipratropium reduces nasal mucus secretion during common cold
(Tagaya et al., 2016)	Tiotropium 18 µg/day, inhaled	Saccharin test; sputum dry/wet ratio; mucin quantification	Reduced sputum solid fraction, mucin content, and nasal mucociliary clearance time	Tiotropium improves sputum characteristics and mucociliary clearance in COPD
(Wang et al., 2019)	Ipratropium bromide 1 mg, inhaled (nebulized)	Bronchoscopic grading of bronchial secretions	Lower bronchial secretion grade during bronchoscopy	Ipratropium nebulization reduces bronchial secretions during bronchoscopy
(Mutmainnah et al., 2023)	Ipratropium bromide 1 mg, inhaled (nebulized)	Bronchoscopic secretion grading; Borg scale; VAS for cough	Lower secretion grade and cough scores compared with control	Inhaled ipratropium reduces cough and bronchial secretions during bronchoscopy

AUC, area under the curve; COPD, chronic obstructive pulmonary disease.

### 3.3 Effect on mucus volume and properties

Four studies directly evaluated secretion volume or the physical characteristics of mucus. In the trials by Taylor et al., 1986 and Pavia et al., 1979, treatment with ipratropium bromide was not associated with significant increases in sputum weight expectorated at 6 or 24 hours, nor with changes in sputum viscosity compared with placebo. In the study by Pavia et al., 1979, a reduction in total sputum weight was observed, without alterations in global mucociliary clearance.

In the study by Tagaya et al., 2016, treatment with tiotropium for eight weeks was associated with a significant reduction in the solid fraction of sputum, as well as a decrease in mucin content (MUC5AC and MUC5B). In turn, Gaffey et al., 1988 demonstrated that intranasal ipratropium partially reduced total nasal mucus production in volunteers with experimentally induced common cold, without modifying the course of the viral infection (**Table 3**).

**Table 3:** Secretion-related outcomes, mucociliary clearance, cough, and safety of inhaled anticholinergics.

Study	Intervention (dose/route/device)	Volume and properties of secretions	Mucociliary clearance	Cough	Safety
(Taylor et al., 1986)	Ipratropium bromide, 200 µg every 8 h (~600 µg/day), inhaled (MDI), 28 days	No differences in sputum weight at 6 h or 24 h; sputum viscosity unchanged; sputum radioactivity similar to placebo	No changes in AUC 0–6 h or corrected AUC; increased peripheral aerosol penetration; alveolar deposition unchanged	No significant differences in cough frequency	Occasional dry mouth; rare bronchospasm; no serious adverse events
(Pavia et al., 1979)	Ipratropium bromide, 40 µg, inhaled (MDI), four times daily for 7 days	Reduced sputum weight; sputum radioactivity unchanged	No change in global mucociliary clearance; increased peripheral aerosol penetration	No clinically relevant changes	Well tolerated; no impairment of mucociliary clearance
(Bennett et al., 1993)	Ipratropium bromide, inhaled (MDI), standard therapeutic dose, single administration	Not directly assessed	Basal mucociliary clearance unchanged	Reduced cough clearance efficiency for radiolabeled particles	No serious adverse events reported
(Hasani et al., 2004)	Tiotropium, 18 µg/day, inhaled (dry powder, HandiHaler^®^)	No adverse changes in airway secretions observed	Increased peripheral aerosol penetration; mucociliary clearance not delayed	No clinically relevant changes	Favorable safety profile; no impairment of mucociliary transport
(Gaffey et al., 1988)	Ipratropium bromide, 84 µg per dose, intranasal spray	Partial reduction in total nasal mucus production over 5 days	Not assessed	Reduced rhinorrhea; no relevant changes in other nasal symptoms	Mild local adverse effects; no serious adverse events
(Tagaya et al., 2016)	Tiotropium, 18 µg/day, inhaled (HandiHaler^®^), 8 weeks	Reduced sputum solid fraction and decreased mucin content (MUC5AC/MUC5B)	Shortened nasal mucociliary clearance time (saccharin test)	Improved cough and sputum scores (CASA-Q)	No clinically relevant adverse events reported
(Wang et al., 2019)	Ipratropium bromide, 1 mg, inhaled (nebulized), single preprocedural dose	Lower bronchial secretion grade during bronchoscopy	Not assessed	Improved patient comfort during the procedure	Higher incidence of transient hypertension; no serious adverse events
(Mutmainnah et al., 2023)	Ipratropium bromide, 1 mg, inhaled (nebulized), single dose	Lower bronchial secretion grade during bronchoscopy	Not assessed	Reduced cough and dyspnea during the procedure	No serious adverse events reported

MDI, metered-dose inhaler; AUC, area under the curve; CASA-Q, Cough and Sputum Assessment Questionnaire.

### 3.4 Mucociliary clearance

The effect of inhaled anticholinergics on mucociliary clearance was primarily evaluated using radioaerosol techniques and functional tests. In the studies by Taylor et al., 1986; Pavia et al., 1979, and Hasani et al., 2004, treatment with ipratropium or tiotropium was not associated with impairment of global mucociliary clearance, as measured by the area under the curve or pulmonary retention of radioaerosols.

Several studies reported an increase in the peripheral penetration index of the aerosol without slowing of mucociliary transport (Pavia et al., 1979; Taylor et al., 1986; Hasani et al., 2004). In addition, Tagaya et al., 2016 observed a significant reduction in nasal mucociliary clearance time, assessed using the saccharin test, following treatment with tiotropium (**Table 3**).

### 3.5 Secretion-related cough

Cough was assessed using different methodological approaches. Bennett et al., 1993 observed that inhaled ipratropium was associated with a reduction in cough effectiveness for the clearance of radiolabeled particles, without changes in baseline mucociliary clearance. Taylor et al., 1986 found no significant differences in the number of cough episodes after four weeks of treatment with ipratropium.

In procedural settings, Wang et al., 2019 and Mutmainnah et al., 2023 reported a significant reduction in cough during bronchoscopy in patients who received nebulized ipratropium as premedication. Likewise, Tagaya et al., 2016 documented clinically relevant improvements in the cough and sputum domains of the CASA-Q questionnaire following treatment with tiotropium (**Table 3**).

### 3.6 Related pulmonary function

Pulmonary function was evaluated as a secondary outcome in several studies. Pavia et al., 1979; Taylor et al., 1986; Hasani et al., 2004; and Tagaya et al., 2016 reported significant improvements in FEV1 after the administration of inhaled anticholinergics, with no evidence of concomitant functional deterioration associated with changes in bronchial secretions (**Table 3**).

### 3.7 Safety and adverse events

Adverse events associated with the use of inhaled anticholinergics were evaluated in several studies. The most frequently reported events corresponded to mild or occasional dry mouth (Taylor et al., 1986), while serious adverse events were infrequent or not reported. Hasani et al., 2004; Tagaya et al., 2016; and Mutmainnah et al., 2023 described no clinically relevant adverse events associated with treatment.

In the study by Wang et al., 2019, a higher incidence of transient hypertension was observed in the ipratropium-treated group during bronchoscopy, without major clinical consequences reported. No adverse events related to impairment of mucociliary clearance or pathological accumulation of secretions were described in the included studies (**Table 3**).

### 3.8 Risk of bias of the included studies

**Figure 2:**
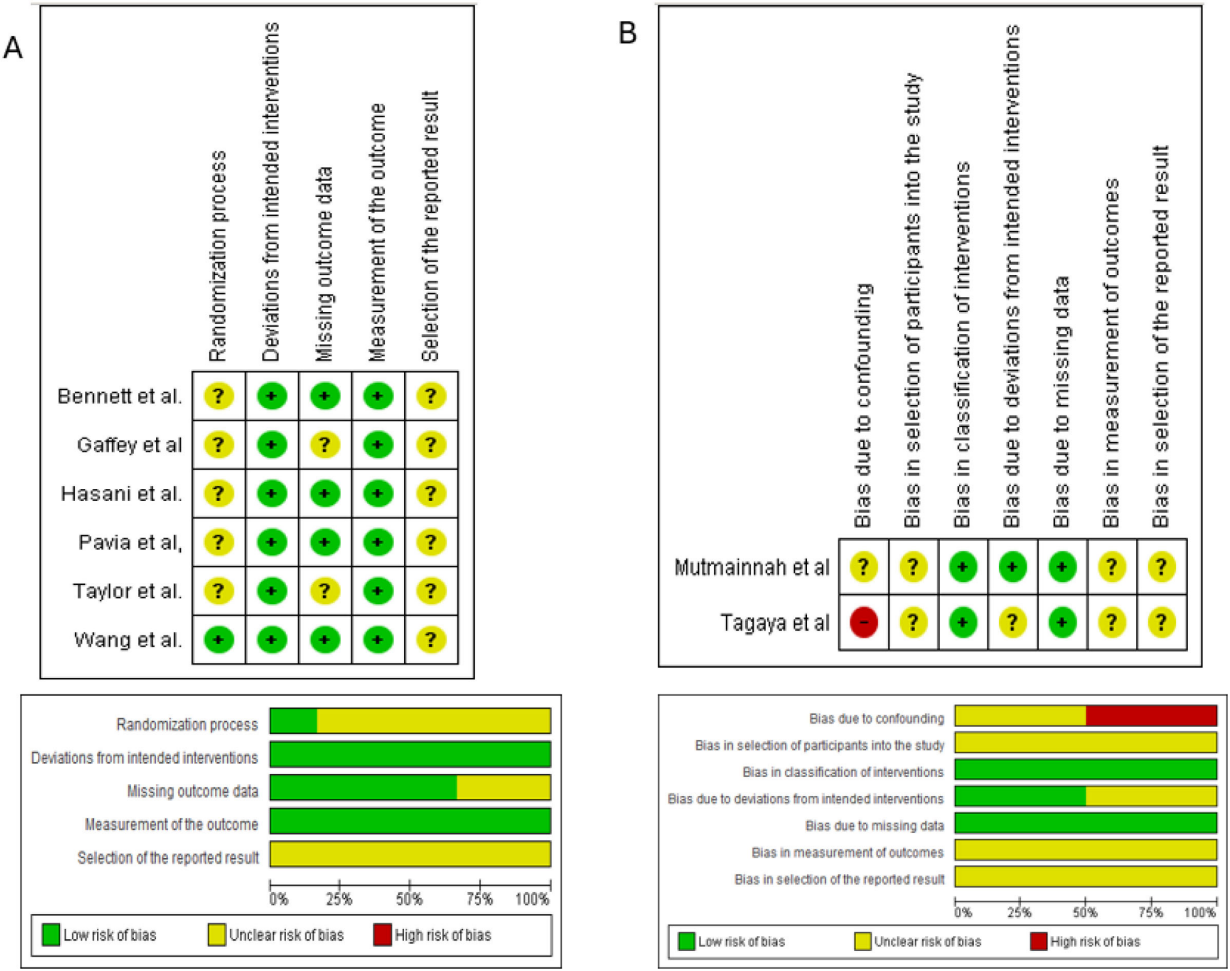
Risk of bias assessment of the included studies. **(A)** Risk of bias of randomized controlled trials assessed using the Cochrane Risk of Bias 2 (RoB 2) tool. **(B)** Risk of bias of non-randomized studies assessed using the ROBINS-I tool.

The risk of bias of the included studies is presented in **Figure 2**. Randomized clinical trials were assessed using the Cochrane Risk of Bias 2 (RoB 2) tool (**Figure 2A**). The studies showed an overall risk of bias classified as low to unclear, with no trials identified as having a high overall risk of bias. The domain related to the randomization process was predominantly classified as unclear risk, whereas domains related to deviations from intended interventions, missing outcome data, and outcome measurement were mostly judged as low risk of bias.

Non-randomized studies were assessed using the ROBINS-I tool (**Figure 2B**). The overall risk of bias was classified as high in the study by Tagaya et al., 2016, while the study by Mutmainnah et al., 2023 presented an overall unclear risk of bias, with individual domains classified between low and unclear risk.

## 4 Discussion

This systematic review examined the effect of inhaled or nebulized anticholinergics on bronchial secretions, particularly their potential influence on the balance between mucus production and airway clearance. Because these drugs act on the cholinergic system, which regulates both bronchial tone and glandular secretion, their effects should be interpreted within the physiological and pathophysiological framework governing mucus handling in the respiratory tract.

From a pharmacological perspective, anticholinergics exert mucoregulatory effects by inhibiting parasympathetic cholinergic activity, a key stimulus for airway mucus secretion (Hocquigny et al., 2024b). This response is mediated mainly by submucosal glands expressing muscarinic M3 receptors, whose activation promotes mucus production. Agents such as ipratropium block these reflexes and may reduce glandular output in conditions of hypersecretion without significantly altering basal secretion volume or mucus viscosity, suggesting modulation of excessive secretion rather than mucus dehydration (Leff, 1996).

Along the bronchial tree, muscarinic receptors M1, M2, and M3 have distinct functions (Alagha et al., 2014). Activation of M1 and M3 receptors promotes bronchoconstriction, airway remodeling, inflammation, and mucus secretion, whereas presynaptic M2 receptors act as inhibitory autoreceptors that limit acetylcholine release. Pharmacologically, greater selectivity for M1 and M3 receptors while preserving M2 function is considered advantageous (Buels and Fryer, 2012). In the proximal airways, mucus is produced by goblet cells and submucosal glands, whereas in the small airways secretion depends mainly on goblet cells, whose increase in COPD contributes to mucus accumulation and small airway disease (Turner et al., 2011). Although long-acting anticholinergics are not direct mucoactive agents, their interference with vagal and non-neuronal cholinergic signaling can indirectly modulate airway mucus secretion (Calzetta et al., 2022).

Based on this framework, it is relevant to examine how these physiological principles translate into the outcomes observed in clinical studies evaluating inhaled anticholinergics in different respiratory contexts, particularly regarding potential changes in mucus volume, composition, clearance, and related clinical outcomes.

From a physiological perspective, acetylcholine acts as a key stimulus for airway mucus secretion through activation of muscarinic M3 receptors located mainly in submucosal glands and, to a lesser extent, in epithelial goblet cells (Belmonte, 2005). However, under basal conditions, mucus production is influenced not only by cholinergic tone but also by inflammatory, structural, and mechanical factors specific to each respiratory disease. Consequently, cholinergic blockade induced by inhaled anticholinergics is unlikely to substantially reduce total secretion production when mucus hypersecretion is driven by chronic inflammation, glandular hyperplasia, or airway remodeling, as occurs in COPD or persistent asthma (Gosens et al., 2006; Ramos et al., 2014).

Although inhaled anticholinergics were not associated with a sustained reduction in secretion production under basal conditions or in chronic clinical scenarios (Pavia et al., 1979; Taylor et al., 1986; Hasani et al., 2004), several studies included in this review described a transient decrease in mucus volume in acute or induced situations (Gaffey et al., 1988; Wang et al., 2019; Mutmainnah et al., 2023). This effect was more evident when hypersecretion was directly mediated by cholinergic stimulation. In contexts of acute airway activation, such as irritative stimulation during endoscopic procedures or early phases of viral infections, blockade of muscarinic M3 receptors was associated with lower secretion production, consistent with direct inhibition of acetylcholine-dependent glandular secretion. Conversely, when mucus production is sustained by chronic inflammatory processes, glandular hyperplasia, or structural airway remodeling, the impact of cholinergic blockade is limited, which helps explain the differences observed between acute effects and findings reported under stable clinical conditions.

In patients with bronchial hypersecretion, inhaled anticholinergics, according to our findings, do not consistently reduce the total amount of secretions produced nor induce a clinically relevant “drying” effect under basal conditions or during chronic treatment. The available evidence indicates that these drugs do not impair mucociliary clearance nor produce relevant changes in mucus rheological properties in stable clinical situations. In this context, modifications in mucus viscosity and viscoelasticity appear to depend mainly on specific mucoactive drugs, such as mucolytics and mucokinetics, which act more directly on the structure of the mucus gel and its macromolecular components (Rhee et al., 1999).

By contrast, in scenarios of acute airway stimulation, some studies suggest that inhaled anticholinergics may transiently modulate cholinergic activation–mediated hypersecretion, particularly through blockade of muscarinic M3 receptors in the submucosal glands. Additionally, in the context of prolonged treatment, a possible effect on mucus composition rather than on its total volume has been described. In the study by Tagaya et al., chronic use of tiotropium was associated with a reduction in mucin content and the solid fraction of sputum, suggesting an indirect modulation of mucin synthesis or secretion processes, without necessarily implying direct changes in viscosity or overall rheological properties of mucus in patients with stable chronic respiratory disease (Tagaya et al., 2016).

The reviewed studies do not demonstrate a consistent direct effect of inhaled anticholinergics on mucociliary transport. In trials employing objective measurements using radioaerosol techniques, such as those by Pavia et al., Bennett et al., and Hasani et al., no deterioration of clearance nor sustained improvement attributable to changes in mucus production or rheological properties was observed (Pavia et al., 1979; Bennett et al., 1993; Hasani et al., 2004). When signals of improved secretion elimination were observed, these appeared to be mainly related to the bronchodilator effects of anticholinergics, which increase airway caliber, reduce airflow resistance, and facilitate distal ventilation, rather than to a direct modification of mucus viscosity, elasticity, or quantity, as also suggested by Taylor et al (Taylor et al., 1986). In this context, experimental data from animal models complement this argument, showing that drugs such as ipratropium and oxitropium do not depress basal mucociliary clearance, whereas atropine may inhibit it under certain conditions and at high concentrations, reinforcing the concept that inhaled anticholinergics used in clinical practice are not associated with a clinically relevant deterioration of mucociliary clearance (Miyata et al., 1989).

With regard to cough, the included evidence is heterogeneous and does not demonstrate a consistent and sustained effect of inhaled anticholinergics under stable clinical conditions. In the studies by Taylor et al. and Hasani et al., treatment with ipratropium or tiotropium was not associated with relevant changes in cough frequency nor with worsening of symptoms (Taylor et al., 1986; Hasani et al., 2004). In contrast, in acute or procedural contexts such as bronchoscopy, Wang et al. and Mutmainnah et al. reported a significant reduction in cough following administration of nebulized ipratropium as premedication (Wang et al., 2019; Mutmainnah et al., 2023). In isolation, the physiological study by Bennett et al. showed a decrease in cough effectiveness for particle clearance without alteration of basal mucociliary clearance, suggesting that effects on cough depend on the clinical context and the outcome evaluated, rather than on a direct impact on mucus production or properties (Bennett et al., 1993). In line with this evidence, clinical guidelines for cough management indicate that long-acting anticholinergics may be useful only in patients with bronchiectasis, and their use should be evaluated according to clinical characteristics and disease severity (Lai et al., 2018).

In contrast to the findings observed in the lower airway, evidence from the upper airway provides an informative physiological comparison. Studies evaluating intranasal anticholinergics have consistently shown a reduction in secretion production, particularly rhinorrhea. For example, in the study by Gaffey et al., intranasal ipratropium was associated with a significant reduction in nasal secretion volume in the context of induced viral infection without affecting other mucosal defense mechanisms. These findings suggest that nasal secretion is more directly regulated by cholinergic stimulation, which may explain why muscarinic blockade produces a clearer secretory effect in the upper airway than in the lower airway, where mucus production is more strongly influenced by inflammatory and structural factors.

A relevant aspect identified in this review is that several studies reporting changes in secretion production used anticholinergic doses higher than those commonly employed in current clinical practice, which may have influenced the observed effects. Typically, in patients with COPD, recommended doses of inhaled anticholinergics are 36 µg of ipratropium via metered-dose inhaler (or 500 µg per nebulization, four times daily), 200 µg of oxitropium two to three times daily, and 5 µg/day of tiotropium via dry powder inhaler (Respimat^®^ Soft Mist), considered safe and effective for chronic disease management (Sharafkhaneh et al., 2013). In the studies included in this review, Hasani et al. used 18 µg/day of tiotropium via dry powder capsule (HandiHaler^®^ dry powder), and Wang et al. and Mutmainnah et al. administered nebulized ipratropium at 1 mg, clearly exceeding usual doses, particularly when used as premedication or in acute contexts such as bronchoscopy. These higher doses were associated with reductions in secretions or cough, suggesting that effects on bronchial secretions may be dose- and route-dependent, and that observed results may not be extrapolated to routine use of standard doses in patients with chronic respiratory disease.

Regarding safety, the included studies did not report serious adverse events associated with the use of inhaled or nebulized anticholinergics. Observed side effects were predominantly local and mild, such as oral or nasal dryness, without relevant clinical impact or treatment discontinuation. Although these effects have been reported in early studies such as Taylor et al. (1986), the evidence is relatively dated and should be interpreted in the context of more recent safety data from larger clinical trials. No increase in bronchospasm nor deterioration of mucociliary clearance attributable to the drug was observed. These findings are consistent with previous evidence demonstrating that inhaled anticholinergics, including long-acting agents such as tiotropium, have a favorable safety profile when used chronically in patients with obstructive respiratory diseases (Tashkin et al., 2008). Nevertheless, considering the high doses used for secretion management, other complex adverse reactions in specific populations should be taken into account. An increased risk of acute urinary retention has been observed, particularly in men with benign prostatic hyperplasia or when drugs are administered via nebulization (Afonso et al., 2011), and evidence regarding cardiovascular effects is heterogeneous. Long-term controlled trials such as UPLIFT indicate that tiotropium does not significantly increase serious cardiovascular events and may even reduce all-cause mortality, although other studies show variable results, suggesting that individual risk factors should be considered and close clinical monitoring performed in patients with cardiovascular comorbidities (Hilleman, 2009).

A limitation of this review is the heterogeneity of the populations included in the analyzed studies. The available evidence encompasses diverse clinical contexts, including patients with asthma, COPD, individuals undergoing bronchoscopy, and healthy volunteers. Although this diversity reflects the limited body of literature specifically addressing the effects of inhaled anticholinergics on airway secretions, it also limits the direct comparability of findings and the ability to draw disease-specific conclusions. In addition, some results may have been influenced by potential confounding factors, such as differences in inflammatory status, concomitant medications, or smoking exposure across study populations. For this reason, results were interpreted according to the clinical context in which they were obtained, distinguishing between acute experimental settings and stable chronic respiratory diseases.

One of the main strengths of this systematic review is that it represents, to our knowledge, one of the first comprehensive synthesis of the effects of inhaled and nebulized anticholinergics on bronchial secretions. Unlike previous studies, this work addresses not only mucus volume, but also rheology, mucociliary clearance, cough, pulmonary function, and safety profile. Given that the use of these drugs in obstructive diseases is often accompanied by the clinical perception—albeit poorly substantiated—that they may “dry” or hinder expectoration, this review provides a critical perspective based on objective measurements and validated risk-of-bias assessment tools. Although heterogeneity in doses and measurement methods limits comparability, this impact was mitigated by prioritizing usual doses and a stratified interpretation by clinical context.

From a clinical perspective, the findings of this review help clarify a common perception in respiratory practice: the concern that inhaled anticholinergics may “dry” airway secretions and impair expectoration. Overall, the available evidence does not support a consistent reduction in mucus production nor a deterioration of mucociliary clearance associated with these agents under stable clinical conditions. These findings suggest the need to reconsider the widely held clinical perception that anticholinergics produce a clinically relevant “drying” of respiratory secretions. Consequently, the presence of bronchial hypersecretion alone should not be regarded as a reason to avoid inhaled anticholinergics when these drugs are otherwise indicated for bronchodilation in obstructive airway diseases. Nevertheless, given the limited number of studies and their methodological heterogeneity, these conclusions should be interpreted with caution.

Future research should focus on well-designed and adequately powered clinical trials that specifically evaluate the effects of inhaled anticholinergics on bronchial secretions across different patient phenotypes, particularly those with marked chronic mucus hypersecretion. Such studies should incorporate standardized and objective measurements of secretion volume, rheological properties, and mucus composition, together with functional outcomes such as cough effectiveness and mucociliary clearance. Longitudinal designs would also help clarify potential long-term effects on mucus biology and their relationship with symptoms, exacerbations, and quality of life, thereby strengthening the evidence base supporting the rational use of inhaled anticholinergics in patients with a high secretion burden. In addition, most available evidence derives from adult populations; therefore, future studies should also explore these effects in pediatric patients, in whom anticholinergics are frequently used in respiratory diseases but their impact on airway secretions remains poorly characterized.

## 5 Conclusion

Inhaled and nebulized anticholinergics do not significantly modify the volume or the properties of bronchial mucus under stable clinical conditions. No deterioration of mucociliary clearance nor relevant alterations in pulmonary function associated with their use were observed.

In situations of acute cholinergic stimulation, such as during bronchoscopic procedures, a transient reduction in secretion production and cough was observed. In chronic respiratory diseases characterized by persistent inflammation or airway remodeling, the effect on hypersecretion was limited.

The included studies did not report serious adverse effects or clinically relevant alterations attributable to the use of inhaled or nebulized anticholinergics. Overall, the findings demonstrate a favorable safety profile and the absence of a negative impact on bronchial secretion clearance mechanisms.

## Data Availability

The original contributions presented in the study are included in the article/supplementary material. Further inquiries can be directed to the corresponding author.
