# Correction: Effect of inhaled anticholinergics on bronchial secretions: a systematic review

**DOI:** 10.3389/fphys.2026.1883894

**Published:** 2026-05-26

**Authors:** Leonardo Arzayus-Patiño, José Julián Bernal-Sanchez, Valeria Pérez-Hortua, Esther Cecilia Wilches-Luna, Vicente Benavides-Cordoba

**Affiliations:** 1 Facultad de Salud, Programa de Fisioterapia, Universidad Santiago de Cali, Cali, Colombia; 2 Grupo de Investigación Salud y Movimiento, Universidad Santiago de Cali, Cali, Colombia; 3 Facultad de Salud, Escuela de Rehabilitación Humana, Cali, Colombia; 4 Grupo de Investigación Ejercicio y Salud Cardiopulmonar (GIESC), Cali, Colombia; 5 Facultad de Salud, Departamento de Ciencias Fisiológicas, Universidad del Valle, Cali, Colombia

**Keywords:** bronchial secretions, cholinergic antagonists, inhaled anticholinergics, mucociliary clearance, sputum

An incorrect **Funding** statement was provided in the published article. The **Funding** statement was published as:

“The author(s) declare that financial support was received for the research and/or publication of this article. This research has been funded by Dirección General de Investigaciones of Universidad Santiago de Cali, under call No. DGI-01–2025 and with the project code (442-621123-385).”

The correct **Funding** statement reads:

“The author(s) declare that financial support was received for the research and/or publication of this article. This research has been funded by Dirección General de Investigaciones of Universidad Santiago de Cali under call No. DGI-01–2026 and project (442-621123-385).”

The original version of this article has been updated.

